# Major cardiovascular events in first-degree relatives of individuals with elevated plasma lipoprotein(a): a registry-based cohort study

**DOI:** 10.1093/eurheartj/ehaf677

**Published:** 2025-08-31

**Authors:** Gustav Kindborg, Daniel Eriksson Hogling, Henrike Häbel, Jane Yan, Teresa Hallerbäck, Örjan Lindhe, Emil Hagström, Daniel P Andersson, Karin Littmann, Jonas Brinck

**Affiliations:** Department of Medicine Huddinge, Karolinska Institutet, Endokrinexpeditionerna C2:94, Karolinska Universitetssjukhuset Huddinge, 141 86 Stockholm, Sweden; Department of Medicine Huddinge, Karolinska Institutet, Endokrinexpeditionerna C2:94, Karolinska Universitetssjukhuset Huddinge, 141 86 Stockholm, Sweden; Department of Learning, Informatics, Management and Ethics, Karolinska Institutet, Stockholm, Sweden; Institute of Environmental Medicine, Department of Medical Epidemiology and Biostatistics, Karolinska Institutet, Stockholm, Sweden; Section of Cardiovascular medicine, Novartis Sverige AB, Stockholm, Sweden; Section of Cardiovascular medicine, Novartis Sverige AB, Stockholm, Sweden; Department of Medical Sciences, Cardiology, Uppsala University, and Uppsala Clinical Research Centre, Uppsala, Sweden; Department of Medicine Huddinge, Karolinska Institutet, Endokrinexpeditionerna C2:94, Karolinska Universitetssjukhuset Huddinge, 141 86 Stockholm, Sweden; Department of Medicine Huddinge, Karolinska Institutet, Endokrinexpeditionerna C2:94, Karolinska Universitetssjukhuset Huddinge, 141 86 Stockholm, Sweden; Department of Medicine Huddinge, Karolinska Institutet, Endokrinexpeditionerna C2:94, Karolinska Universitetssjukhuset Huddinge, 141 86 Stockholm, Sweden

**Keywords:** Lipoprotein(a), Atherosclerotic cardiovascular disease, Major adverse cardiovascular event, Primary prevention, Screening

## Abstract

**Background and Aims:**

Lipoprotein(a) [Lp(a)] levels are genetically determined and causal in the development of atherosclerotic cardiovascular disease. Whether first-degree relatives (FDRs) of individuals with elevated Lp(a) levels (≥80th percentile) have an increased cardiovascular disease risk is unknown.

**Methods:**

Based on 41 304 indexes with a routine plasma Lp(a) measurement, 61 715 FDRs without a measured Lp(a) aged 35–69 years (49% women) were identified in the Swedish STRIREG cohort. First-degree relatives were stratified according to index Lp(a) percentile level: <50th, 50–<80th, 80–<95th, and ≥95th. In competing risk-adjusted cumulative incidence and adjusted Cox proportional hazards regressions, the association between Lp(a) strata and major adverse cardiovascular events (MACE; cardiovascular death, myocardial infarction, ischaemic stroke, coronary revascularization) was investigated. In a nested analysis of 4243 indexes with a first-degree relationship with another index, the concordance of plasma Lp(a) levels was assessed.

**Results:**

During a median follow-up of 19 (11–26) years, 2043 FDRs had a MACE. The cumulative incidences of MACE in FDR until age of 65 were 6.2%, 7.0%, 7.5%, and 8.1% by increasing index Lp(a) level strata (*P* < .001). Compared with FDR in the lowest Lp(a) stratum, there was a higher hazard ratio for MACE by increasing Lp(a) stratum: 1.08 (95% confidence interval, 0.97–1.19), 1.30 (1.15–1.47), and 1.28 (1.06–1.55; *P*_trend_ < .001). The Lp(a) concordance was 86% (<80th percentile) and 53% (≥80th percentile).

**Conclusions:**

First-degree relatives of individuals with elevated Lp(a) levels have a higher incidence of MACE. Cascade screening could be a feasible strategy to identify FDR at heightened risk.

## Structured Graphical Abstract

**Structured Graphical Abstract:**
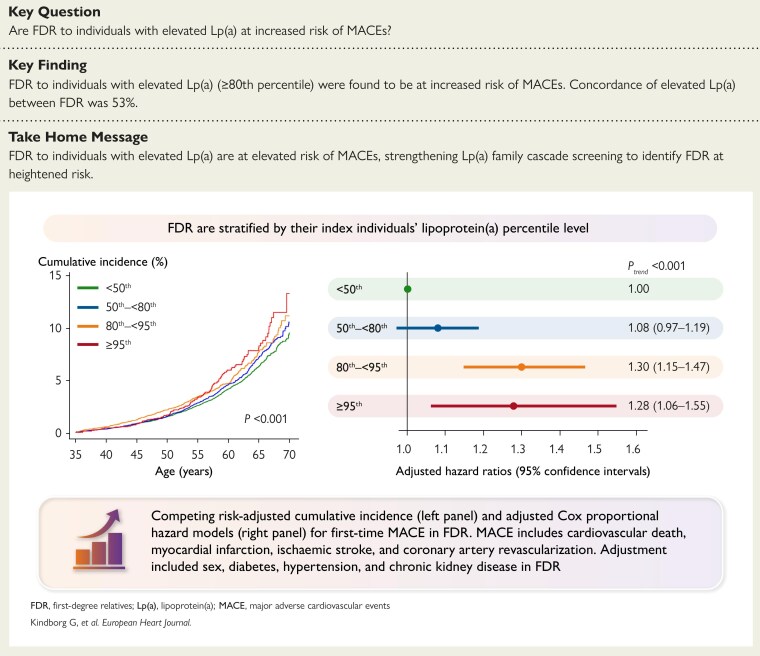
FDR, first-degree relative; MACE, major adverse cardiovascular event.

**See the editorial comment for this article ‘Cascade screening for high lipoprotein(a): the time has come’, by V. Bittner, https://doi.org/10.1093/eurheartj/ehaf830.**

## Introduction

Lipoprotein(a) [Lp(a)] is causal in the development of major ischaemic events affecting predominantly the coronary arteries,^1–3^ and the cardiovascular risk conferred by Lp(a) is independent of traditional risk factors including LDL cholesterol levels.^4–6^ The atherogenicity of Lp(a) has been reported to be substantially greater per particle than that of LDL.^7,8^ Plasma Lp(a) levels predict development of atherosclerotic cardiovascular diseases in a dose-dependent fashion when measured in children or middle-aged individuals.^9–12^ The distribution of Lp(a) in plasma is highly skewed in the population, with most individuals having relatively low levels. A plasma concentration of above 120 nmol/L (or 50 mg/dL), corresponding to the 80th percentile in the general population, is associated with an increased relative lifetime risk for major ischaemic events including both myocardial infarction and ischaemic stroke of approximately 30% compared with those with a median (20 nmol/L) level.^1^ An elevated Lp(a) is also associated with increasing risk of recurrent atherosclerotic cardiovascular events.^13^

An individual’s plasma Lp(a) concentration is predominantly determined by genetics, with only a modest contribution of lifestyle factors, comorbidities, and medications.^14^ The *LPA* gene, coding for apolipoprotein(a), has a complex structure including multiple functional single nucleotide polymorphisms causing Lp(a) concentrations to vary between individuals with similar genetic isoforms.^15,16^ Despite the complexity of the inheritance influencing Lp(a) concentrations, there is substantial concordance in plasma Lp(a) concentrations between family members. The concordance of elevated Lp(a) between first-degree relatives (FDRs) in a general population setting has been reported to be around 50% if the index individual has an Lp(a) level exceeding 125 nmol/L.^17^

Despite data on the hereditary patterns and proatherosclerotic properties of Lp(a), there is a paucity in knowledge on whether elevated Lp(a) in an index individual also confers an increase in cardiovascular disease risk to the FDR. We aimed to determine the risk of atherosclerotic cardiovascular diseases in FDR to indexes with a known Lp(a) value registered in the multigenerational STRIREG (Stockholm hyperTRIglyceridaemia REGister) cohort.^18^ We hypothesized that elevated Lp(a) in indexes is associated with increased cardiovascular disease risk in FDR.

## Methods

### Study design and cohorts

This is an observational register-based cohort study based on the STRIREG database including consecutive individuals with a Lp(a) measurement performed during routine clinical practice between 2000 and 2021, herein termed indexes, and their FDR.^18^ The indication for plasma lipid sampling was determined at the discretion of the treating physician and was performed either during hospitalization or at an outpatient clinic in the county of Stockholm. The FDR cohort was defined as all parents, full siblings, or children to an index but without an Lp(a) measurement. First-degree relatives were identified by linkage using the unique personal identity numbers for indexes and FDR in the Swedish Multi-Generation Register governed by Statistics Sweden. The linkage was performed regardless of whether the FDR was dead or alive. A subset of the indexes had a first-degree relationship to at least one other index. The cohorts are detailed in Supplementary data online, *Figure S1*. Data on demographic characteristics and cardiovascular outcomes in both the index and the FDR were collected from national mandatory registers and included the National Cause of Death Registry (1987–2021), the Inpatient registry (1987–2021), and the Outpatient registry (2001–21). Only FDR born between 1 January 1952 and 1 January 1987 were included. The age restriction was chosen to ensure adequate coverage of participants’ outcomes in the national registers used and allowed for participants to be evaluated for incident cardiovascular outcomes starting at age 35 to a maximum of 69 years. Only birth year of the participants was available in the dataset and the birth date was set to 1 January of the year of birth. Individuals were excluded if they lacked a valid personal identification number or had died before the age of 35 years. The study was approved by Swedish Ethical Review Authority (Dnr 2021-03883) and was performed in accordance with the principles of the Declaration of Helsinki.

### Determination of lipoprotein(a), lipoprotein(a) stratification, and plasma lipoprotein(a) concordance

Lipoprotein(a) was measured at either the Karolinska University Laboratory or the Unilabs Laboratory, both in Stockholm, Sweden, between 1 January 2000 and 31 December 2021. In total, seven Lp(a) laboratory methods were used during the time period to measure Lp(a) in either molar (nmol/L) or mass (mg/dL). The frequency of the methods used and the Lp(a) distributions are described in Supplementary data online, *Tables S1* and *S2* and *Figure S2*. The index cohort was divided into four Lp(a) strata based on Lp(a) percentile distribution in either mass or molar concentrations: <50th, 50th–<80th, 80th–<95th, and ≥95th. These percentiles were selected as they reflect clinically established thresholds for low, intermediate, high, and very high risk.^1,19,20^ For Lp(a) stratification, the earliest available measurement was used unless both molar and mass values were recorded, in which case the first measurement in molar was used. A sensitivity analysis was conducted based internal laboratory analysis codes representing the different Lp(a) assays to assess if changes in Lp(a) assays would reclassify index stratum categorization (see Supplementary data online, *Table S3*). First-degree relatives were categorized into identical four Lp(a) strata based on the Lp(a) value of their corresponding index. If an FDR was associated with multiple indexes, categorization was based on the index whose Lp(a) measurement was recorded first.

Concordance of plasma Lp(a) levels between individuals was determined in a subset of indexes who had a first-degree relationship to another index. Within each unique relationship, the index was defined as the individual having had their Lp(a) value determined first.

### Exposures and outcomes

The study exposure for the FDR was the Lp(a) stratum of their respective index. The study exposure for indexes was their allocated Lp(a) stratum. Study outcomes were major adverse cardiovascular events (MACE), the composite of cardiovascular death, myocardial infarction, ischaemic stroke and coronary revascularization, and the individual components (see Supplementary data online, *Table S4*), based on the International Classification of Diseases diagnosis (ICD-9, ICD-10) and surgical intervention codes.

### Statistical analysis

Analyses were performed in stratum of FDR according to their index Lp(a) stratum. Demographic and clinical characteristics are summarized as median and interquartile range (IQR) for numerical variables and as counts and proportions for categorical variables. Lipoprotein(a) distribution was visualized as percentage proportion on *y*-axis and Lp(a) concentration on *x*-axis for the two different Lp(a) methods used. Cumulative incidence functions in the presence of competing risks presented by non-cardiovascular death were estimated for indexes and FDR aged 35–69 years. Crude differences in cumulative incidence functions between Lp(a) groups were tested using Gray’s test. Incidence rates per 1000 person-years and their 95% confidence intervals (CI) were estimated, and *P*-values using the mid-*P* adjustment for tests of incidence rate difference were reported for pairwise comparisons.

Cox proportional hazards regression models were used to assess the association between exposure categories and outcomes, presented as hazard ratios with 95% CI. The analyses were adjusted for age, sex, diabetes, hypertension, and chronic kidney disease (ever during follow-up). The adjustment variables were chosen based on models derived from previous work (see Supplementary data online, *Figure S3*). Covariates were defined according to ICD-9, ICD-10, and surgical intervention codes (see Supplementary data online, *Table S4*). No information on LDL-cholesterol was available. The lowest Lp(a) stratum (<50th percentile) served as the reference group. The proportional hazard assumption was visually evaluated using Schoenfeld residual and log–log survival plots. Schoenfeld residuals were plotted against time for each covariate to identify any time-dependent effects, while log–log survival plots were used to compare survival probabilities across groups (see Supplementary data online, *Figure S4*).

Interaction effects between a dichotomized Lp(a) level (≥80th percentile compared with <50th percentile) and each covariate for MACE outcomes were separately tested using Wald’s tests on crude and adjusted hazard ratios in FDR. Individual effects were investigated in subgroups defined by the levels of the respective covariates. Heredity for premature disease in FDR was defined as presence of atherosclerotic cardiovascular disease (MACE or peripheral artery disease) in their respective index at age < 55 years for males and age < 60 years for females (see Supplementary data online, *Table S4*).

The concordance of plasma Lp(a) between individuals with a first-degree relationship was expressed as percentage in each Lp(a) stratum and as a relative risk.

All statistical analyses were performed at the Karolinska Institutet, Stockholm, using Stata version 16.1 (StataCorp. 2018. Stata Statistical Software: Release 16. College Station, TX: StataCorp LLC). Competing risk analyses were based on the stcompet command and performed in R including Gray’s test.^21,22^ Two-sided *P*-values were reported, and a *P* < .05 was considered statistically significant. Owing to the explorative nature of this study, no multiple testing adjustments were made, and generated hypotheses are subject to confirmation.

## Results

### Study cohorts

The study included 61 715 FDR without an Lp(a) measurement related to the 41 304 indexes with an Lp(a) measurement (see Supplementary data online, *Figure S1*). The 50th, 80th, and 95th percentiles in the indexes’ Lp(a) distributions in mass and molar concentrations were determined to 20, 114, and 239 nmol/L and 18, 56, and 121 mg/dL, respectively (see Supplementary data online, *Figure S2*). First-degree relatives were allocated to four Lp(a) strata, < 50th, 50th–<80th, 80th–<95th, and ≥95th, based on their respective index’s percentile Lp(a) level.

### First-degree relatives

#### Cardiovascular outcomes

**Figure 1:**
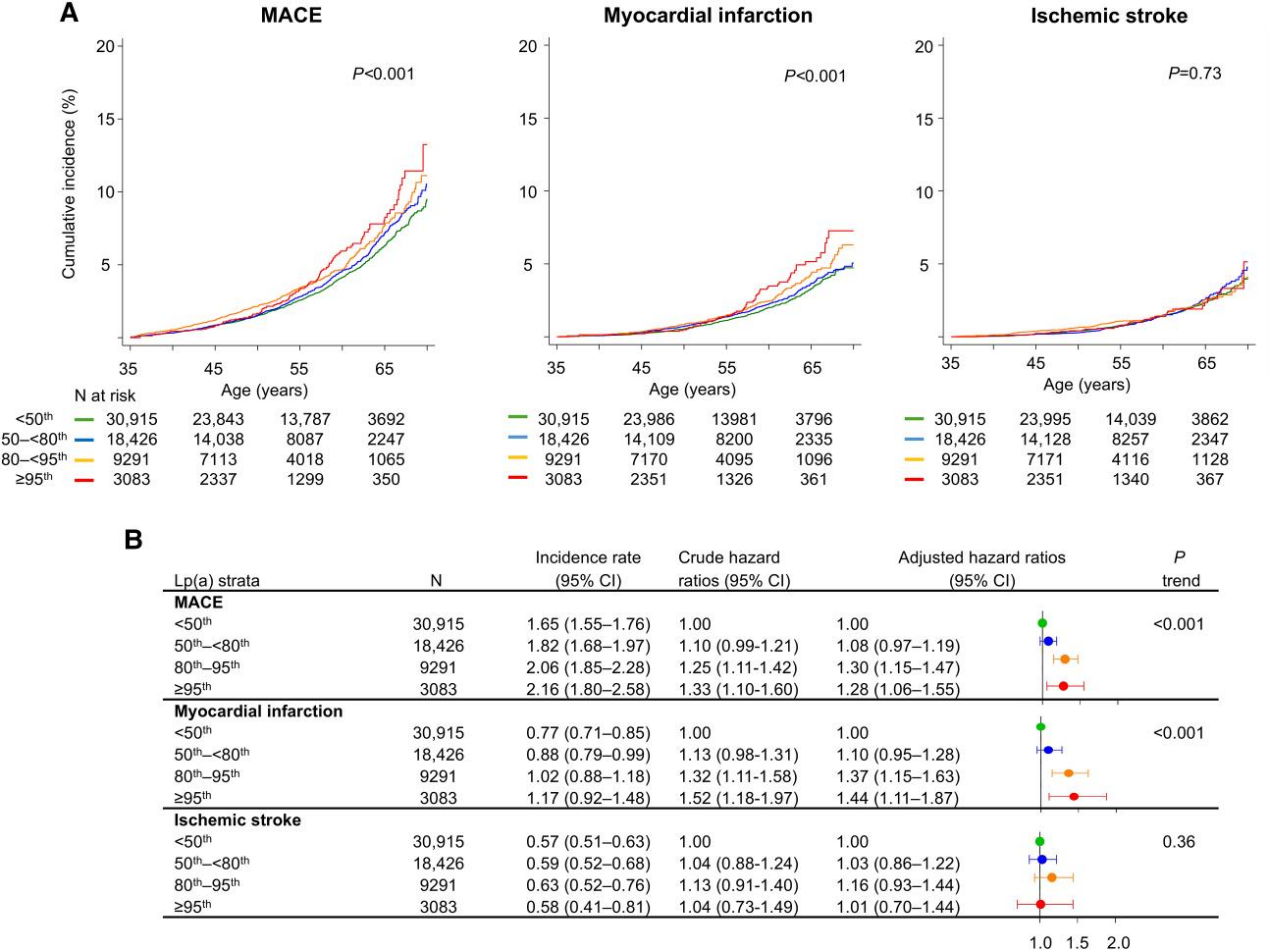
Competing risk-adjusted cumulative incidence (*A*), incidence rates and hazard ratios (*B*) of incident major adverse cardiovascular events in first-degree relatives aged 35–69 years stratified according to index lipoprotein(a) level. Incidence rates were calculated per 1000 person-years. Lowest lipoprotein(a) stratum was used as the reference in the Cox regression model adjusted for sex, diabetes, hypertension, and chronic kidney disease. CI, confidence interval; FDR, first-degree relatives; MACE, major adverse cardiovascular events, including cardiovascular death, myocardial infarction, ischaemic stroke, and coronary artery revascularization

The FDR had a median age of 53 years (IQR 45–60) at the end of follow-up and women constituted 49% (*Table 1*). First-degree relatives were followed for a median of 19 years **(**IQR 11–26) during which 2043 MACE occurred. The cumulative incidence of MACE for FDR by higher index Lp(a) stratum from age 35 years showed curve separation with the highest Lp(a) strata associated with the highest event rate (*P* < .001) (*Figure 1A*). Cumulative incidences until age 65 years were 6.2%, 7.0%, 7.5%, and 8.1% by higher Lp(a) stratum (*P* < .001). Cumulative incidences until age 65 years by higher index Lp(a) stratum were also observed for myocardial infarction, corresponding to 3.2%, 3.6%, 4.2%, and 5.0% (*P* < .001) but not for ischaemic stroke (2.3%, 2.5%, 2.5%, 2.3%; *P* = .73). The incidence rates per 1000 person-years for MACE and myocardial infarction increased by higher Lp(a) stratum but not for ischaemic stroke (*Figure 1A*). Compared with FDR in the lowest index Lp(a) stratum, there was a risk increase for every increase in Lp(a) stratum for MACE (*P*_trend_ < .001) and myocardial infarction (*P*_trend_ < .001) but not for ischaemic stroke (*P*_trend_ = .36) (*Figure 1B*). There were, in total, 633 (1.0%) cardiovascular deaths and 200 (0.3%) incident coronary revascularizations during follow-up. There was no difference in cumulative incidences between FDR in the Lp(a) strata for cardiovascular death (*P* = .27), whereas coronary revascularization increased in FDR with increasing index Lp(a) stratum (*P* = .023). (see Supplementary data online, *Figure S5*). The rate of coronary revascularizations increased during the study period, with a clear trend from year 1997 onwards (see Supplementary data online, *Table S6*).

**Table 1:** Characteristics of first-degree relatives and indexes at end of follow-up

	FDR: Lp(a) percentile group				Total (*N* = 61 715)	Indexes: Lp(a) percentile group				Total (*N* = 17 360)
	<50th (*N* = 30 915)	50th–<80th (*N* = 18 426)	80th–<95th (*N* = 9291)	≥95th (*N* = 3083)		<50th (*N* = 8910)	50th–<80th (*N* = 5197)	80th–<95th (*N* = 2471)	≥95th (*N* = 782)	
Birth year	1967 (1960–75)	1968 (1960–76)	1968 (1961–75)	1968 (1961–76)	1967 (1960–75)	1963 (1957–71)	1963 (1957–71)	1962 (1956–70)	1961 (1955–68)	1963 (1957–71)
Age, years, median (IQR)	53 (46–60)	53 (45–60)	53 (45–60)	53 (45–60)	53 (45–60)	57 (49–64)	58 (50–64)	58 (50–64)	60 (53–65)	58 (50–64)
Female sex, *n* (%)	15 367 (49.7)	9148 (49.6)	4540 (48.9)	1466 (47.6)	30 521 (49.5)	4152 (46.6)	2531 (48.7)	1189 (48.1)	404 (51.7)	8276 (47.7)
Deceased, *n* (%)	990 (3.2)	593 (3.2)	296 (3.2)	100 (3.2)	1979 (3.2)	374 (4.2)	201 (3.9)	97 (3.9)	43 (5.5)	715 (4.1)
MACE, *n* (%)	974 (3.2)	627 (3.4)	354 (3.8)	124 (4.0)	2079 (3.4)	849 (9.5)	595 (11.4)	321 (13.0)	146 (18.7)	1911 (11.0)
Myocardial infarction, *n* (%)	458 (1.5)	304 (1.6)	176 (1.9)	68 (2.2)	1006 (1.6)	467 (5.2)	361 (6.9)	185 (7.5)	99 (12.7)	1112 (6.4)
Ischaemic stroke, *n* (%)	347 (1.1)	211 (1.1)	113 (1.2)	35 (1.1)	706 (1.1)	290 (3.3)	194 (3.7)	97 (3.9)	36 (4.6)	617 (3.6)
Coronary artery revascularization, *n* (%)	86 (0.3)	65 (0.4)	37 (0.4)	16 (0.5)	204 (0.3)	118 (1.3)	103 (2.0)	57 (2.3)	29 (3.7)	307 (1.8)
Hypertension, *n* (%)	3381 (10.9)	2050 (11.1)	987 (10.6)	351 (11.4)	6769 (11.0)	2817 (31.6)	1734 (33.4)	847 (34.3)	306 (39.1)	5704 (32.9)
Heart failure, *n* (%)	386 (1.2)	198 (1.1)	134 (1.4)	44 (1.4)	762 (1.2)	361 (4.1)	249 (4.8)	119 (4.8)	50 (6.4)	779 (4.5)
Diabetes mellitus (any type), *n* (%)	1343 (4.3)	821 (4.5)	410 (4.4)	122 (4.0)	2696 (4.4)	2172 (24.4)	1197 (23.0)	519 (21.0)	160 (20.5)	4048 (23.3)

MACE, major adverse cardiovascular event.

#### Cardiovascular outcomes according to sex

**Figure 2:**
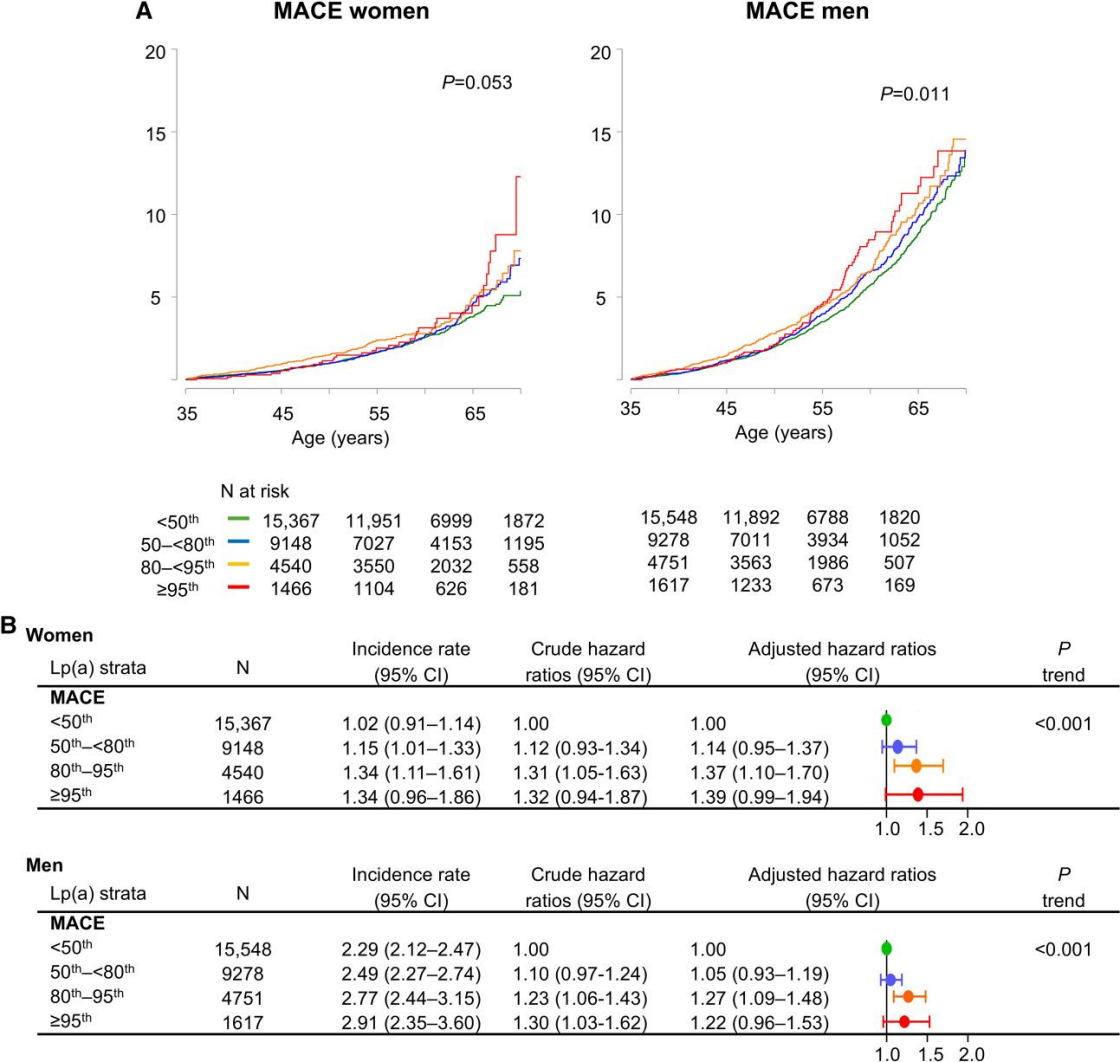
Competing risk-adjusted cumulative incidence (*A*), incidence rates and hazard ratios (*B*) of incident major adverse cardiovascular events in first-degree relatives aged 35–69 years stratified according to their index lipoprotein(a) level and sex. Incidence rates were calculated per 1000 person-years. Lowest lipoprotein(a) stratum was used as the reference in the Cox regression proportional hazard model adjusted for sex, diabetes, hypertension, and chronic kidney disease. CI, confidence interval; FDR, first-degree relatives; MACE, major adverse cardiovascular events, including cardiovascular death, myocardial infarction, ischaemic stroke, and coronary artery revascularization

Cumulative incidences for MACE before age 65 years in female FDR were 3.7%, 4.5%, 4.8%, and 4.4% (*P* = .053) and in male FDR were 8.6%, 9.5%, 10.2%, and 11.4% (*P* = .011) by increasing Lp(a) stratum (*Figure 2A*). The incidence rates were in general approximately double for male FDR compared with female FDR (*Figure 2B*). Increases in adjusted hazard ratios were observed in female FDR (*P*_trend_ = .002) and male (*P*_trend_ = .002) FDR with increasing Lp(a) strata when comparing with the <50th stratum.

#### Major adverse cardiovascular event outcomes and interaction with cardiovascular risk factors

**Figure 3:**
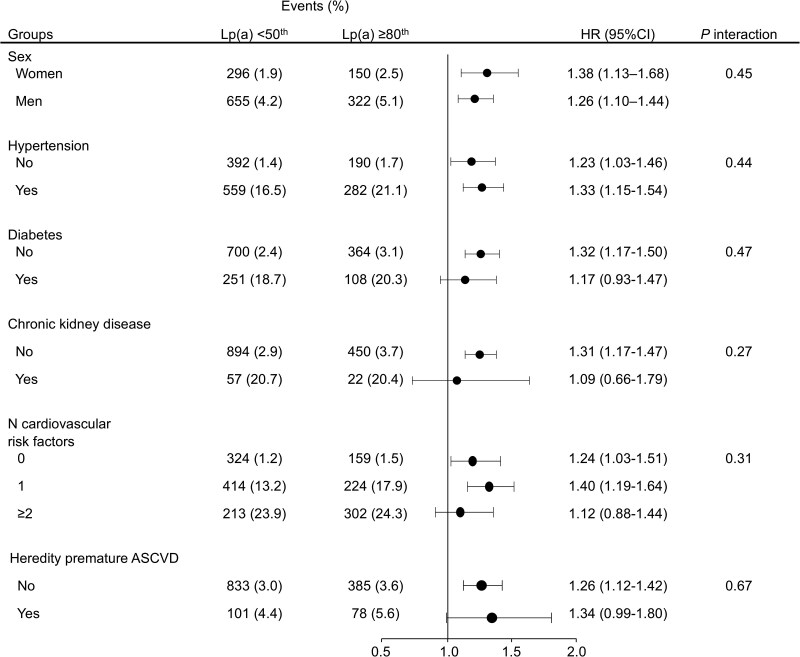
The proportions of and hazard ratios for major adverse cardiovascular events in first-degree relatives in lipoprotein(a) < 50th vs lipoprotein(a) ≥ 80th groups and interaction with risk factors. The presence of risk factors was evaluated at end of follow-up. FDR, first-degree relatives; MACE, major adverse cardiovascular events

The risk for incident MACE was compared between Lp(a) strata < 50th and ≥80th to analyse the potential interactions between sex, cardiovascular risk factors (hypertension, diabetes, chronic kidney disease), and heredity for premature atherosclerotic cardiovascular disease for the outcome (*Figure 3*). The difference in the proportion of events in the various subgroups ranged from 1.2%, in the <50th subgroup without any cardiovascular risk factors and the highest proportion, to 24.3%, in the ≥80th subgroup with ≥2 risk factors. The hazard ratios were consistently higher for Lp(a) ≥ 80th subgroups compared with Lp(a) < 50th subgroups in all analyses. None of the risk factors, separately or in combination, displayed any interaction with the risk for MACE (*P* > .05 for all).

### Indexes

#### Cardiovascular outcomes

**Figure 4:**
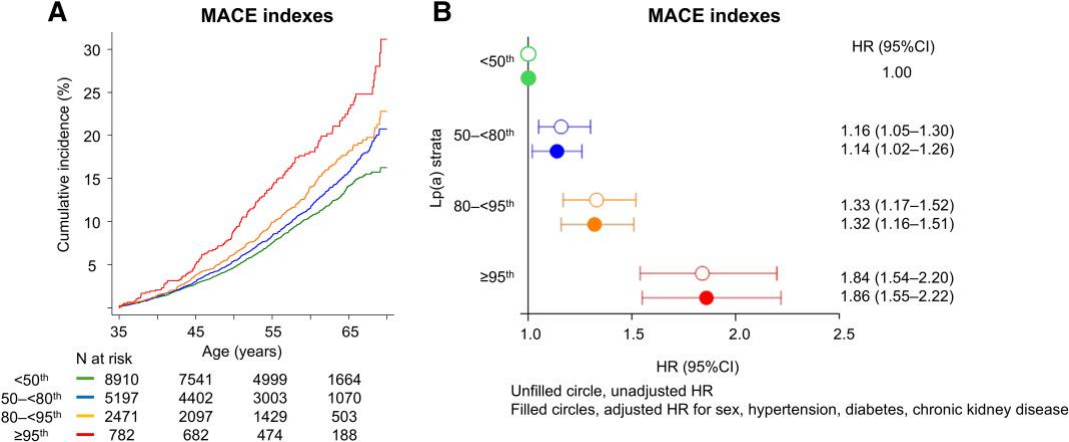
Competing risk-adjusted cumulative incidence (*A*) and hazard ratios (*B*) of incident major adverse cardiovascular events in indexes aged 35–69 years stratified according to their lipoprotein(a) level. Lowest lipoprotein(a) stratum was used as the reference in the Cox proportional hazards regression model. CI, confidence interval; MACE, major adverse cardiovascular events, including cardiovascular death, myocardial infarction, ischaemic stroke and coronary revascularization

A total of 17 360 indexes from age 35 years were assessed for development of cardiovascular outcomes. The median age was 58 years (IQR 50–64) and women constituted 48% (*Table 1*). The indexes were followed for a median 22 years (IQR 19–29) during which 1862 MACE occurred. The cumulative incidence of MACE by higher index Lp(a) stratum from age 35 years showed early curve separation with the highest Lp(a) stratum associated with the highest event rate (*P* < .001) (*Figure 4A*). Cumulative incidences until age 65 years were 13.9%, 15.6%, 17.9%, and 23.0% by higher Lp(a) stratum. The incidence rate (95% CI) per 1000 person-years for MACE increased from 4.38 (4.09–4.69) in the lowest to 8.28 (7.03–9.74) in the highest Lp(a) stratum (see Supplementary data online, *Table S5*). Similarly, the crude and adjusted hazard ratios (95% CI) for MACE increased with increasing Lp(a) stratum (*P*_trend_ < .001) (*Figure 4B*). Further, indexes had a higher cumulative incidence (*P* < .001) and increased hazard ratios for (*P*_trend_ < .001) myocardial infarction by increasing Lp(a) stratum but not of ischaemic stroke (*P* > .05 for both comparisons). Cumulative incidences, incidence rates, and hazard ratios stratified by Lp(a) stratum for the individual MACE components for indexes are compiled in Supplementary data online, *Figure S6*.

### Concordance of plasma lipoprotein(a) between indexes with a first-degree relationship

**Figure 5:**
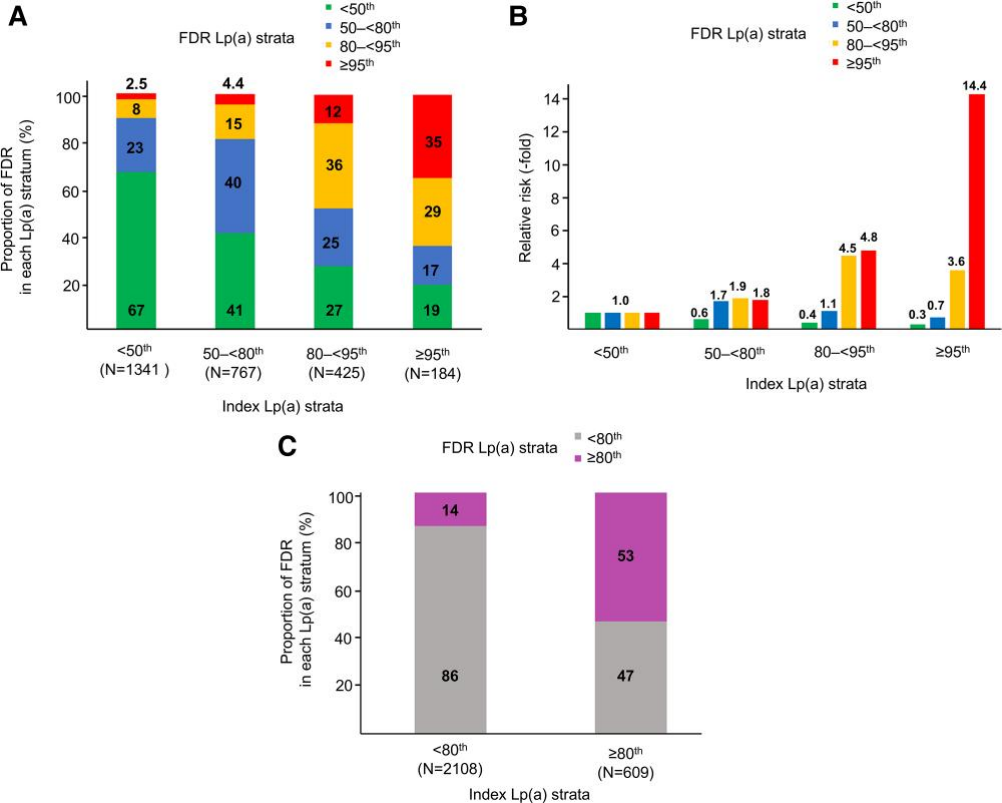
Concordance of plasma lipoprotein(a) levels between 4243 first-degree related (parent, sibling, child) individuals forming 2717 unique relationships. Concordance for each stratum was calculated based on four (*A*) and two (*C*) lipoprotein(a) strata and is indicated with bold numbers. The proportions of Lp(a) between individuals in the other strata are indicated with non-bold numbers. The relative risk (*B*) for an first-degree relative to have lipoprotein(a) < 50th, 50th–<80th, 80th–<95th, or ≥95th percentile in comparisons to first-degree relatives to indexes with lipoprotein(a) < 50th percentile (relative risk = 1)

A total of 4243 indexes had a first-degree relationship to another index forming 2717 unique index–FDR pairs (parent–child, sibling–sibling, child–parent). The concordance of plasma Lp(a) between index–FDR pairs for each Lp(a) stratum was 67% (<50th), 40% (50th–<80th), 36% (80th–<95th), and 35% (≥95th) (*Figure 5A*). The relative risk for an FDR to have Lp(a) ≥ 95th percentile was 4.8-fold and 14.4-fold higher when the index had Lp(a) 80th–<95th or ≥95th percentile, respectively, compared with Lp(a) < 50th (*Figure 5B*). The Lp(a) concordances between index–FDR pairs with an Lp(a) < 80th percentile and an Lp(a) ≥ 80th percentile were 86% and 53%, respectively (*Figure 5C*). The pattern of concordance was similar across all types of first-degree relationships (see Supplementary data online, *Figure S7*).

## Discussion

This observational study investigates the risk of MACE in over 60 000 FDR of individuals with a known Lp(a) level during 35 years of follow-up. There are three key findings. First, the hazard ratio for MACE is approximately 30% higher in FDR to indexes with elevated Lp(a) (above the >80th percentile level, >114 nmol/L, or >56 mg/dL) compared with those with low levels (<50th percentile). Second, the risk increment of cardiovascular events in FDR to indexes with elevated Lp(a) is observed in both women and men and is independent of traditional risk factors. Third, the concordance of elevated Lp(a) between a subgroup of indexes with a first-degree relationship was 53% in a nested analysis (*Structured Graphical Abstract*). Taken together, the study identifies FDR to indexes with elevated Lp(a) as individuals at increased risk of developing cardiovascular disease.

The study quantifies the absolute risk increment for FDR to indexes with an elevated Lp(a) to experience incident MACE to 1.5%–2% until age 65 years compared with FDR to indexes with low Lp(a). The increment observed in FDR is approximately one-third of the indexes’ risk increment (4%–9%) until the same age. The index cohort is likely not to be fully representative of the general population since the indication for their Lp(a) sampling is unknown and was performed during a time period when Lp(a) measurement was not part of the clinical routine. However, it displays a clear resemblance to general population cohorts, with predominantly white populations, with a similar Lp(a) distribution and incidence rates between lowest to highest Lp(a) strata for cardiovascular events.^10,11,23^ The study results indicate that elevated Lp(a) is primarily associated with higher risk for coronary artery disease, including myocardial infarction and coronary revascularization, but not with ischaemic stroke in FDR, which is also consistent with previously reporting that Lp(a) targets mainly the coronary arteries.^24,25^ The relatively low number of coronary revascularizations (percutaneous coronary intervention or coronary artery bypass graft surgery) for FDR in the study can in part be explained by lower revascularization rates in the first part of the observational period when thrombolysis was the dominant intervention for myocardial infarction. There was no association observed between elevated Lp(a) in index and ischaemic stroke in FDR. As cerebrovascular disease has multiple aetiologies beyond atherosclerotic vascular disease,^26^ this may potentially explain the lack of association between elevated Lp(a) and ischaemic stroke.

There was no observed difference in cardiovascular death comparing FDR in different Lp(a) strata, which could be due to the middle-aged population investigated having a relatively low background risk for cardiovascular disease. First-degree relatives of both sexes experience a relative risk increase for MACE in the elevated Lp(a) stratum, but the incidence rate for MACE was about half for female FDR. It is well established that middle-aged women have lower absolute risk for atherosclerotic cardiovascular disease than men, but the risk accelerates at older age. Plasma Lp(a) increases from menopause, and the risk identified in female FDR could therefore increase with increasing age, both in relative and absolute terms.^27^

There was a concordance for an elevated plasma Lp(a) between individuals with a first-degree relationship of approximately 50% in a nested subgroup. The result echoes other studies and underscores the strong heredity of the elevated Lp(a) trait.^17,28,29^ For example, testing for Lp(a) in FDR undergoing cascade screening for familial hypercholesterolaemia and other dyslipidaemias has demonstrated a considerable aggregation of elevated Lp(a) in family members. The increase in MACE cumulative incidence before the age of 65 years for FDR is one-third to that observed in indexes in the Lp(a) stratum 80th–<95th at the same age and one-fifth in indexes in the Lp(a) stratum ≥95th. The lower cumulative incidence of MACE observed in FDR compared with indexes is at a level that is slightly lower than but still comparable to the estimated concordance for elevated Lp(a) within these strata. The combination of the strong genetic regulation of Lp(a) and its high plasma concordance between individuals with a first-degree relationship therefore strongly suggest that the cardiovascular risk increase observed in FDR is at least in part attributable to an inheritance of an elevated plasma Lp(a). Further research based on systematic and prospective collected plasma Lp(a) measurement of FDR is needed to confirm the true heritability of elevated plasma Lp(a).

The study findings give support for an independent role of Lp(a) for the development of MACE. The cardiovascular risk asserted by Lp(a) has been reported to be independent of LDL cholesterol and apolipoprotein B concentrations.^4,5,11^ Based on Lp(a) stratification of their respective index in the study, an FDR with elevated Lp(a) has a relative risk increase regardless of sex, hypertension, or the presence of one risk factor. In FDR with a severely compounded risk for MACE (e.g. FDR with diabetes, chronic kidney disease, or multiple risk factors), the relative impact of Lp(a) stratification is reduced. Lipoprotein(a) is an independent risk factor for the development in patients with diabetes and chronic kidney disease.^30–32^ The weaker association between cardiovascular risk and Lp(a) reported here is most probably explained by the larger impact of FDR’s own risk factors for the development of disease than the risk conferred by a potential inheritance of Lp(a). However, due to small subgroup sizes in the interaction analysis between elevated Lp(a) and cardiovascular risk factors, we cannot exclude the possibility that significant interactions could exist.^33^

The observation that heredity for premature cardiovascular disease is associated with MACE in FDR is interesting. A strong association between elevated Lp(a) and premature disease in the index on one hand and MACE in FDR on the other would imply that Lp(a) is the causal contributor to the development of cardiovascular disease in FDR. However, the absence of this association does not exclude a mediating role for Lp(a), since elevated Lp(a) and a family history of premature myocardial infarction have been reported to have both independent and additive joint associations with cardiovascular risk.^34,35^

The study identifies FDR to indexes with an elevated Lp(a) as individuals at heightened cardiovascular risk *per se*. An individual clinical cardiovascular risk evaluation of these FDR would have the potential to detect risk factors, including an elevated Lp(a), that can be addressed in clinical practice. New drug treatments specifically targeting elevated Lp(a) are also under clinical evaluation that could alleviate cardiovascular risk in the future.^36^ Guidelines and national recommendations for the management of dyslipidaemia recommend plasma Lp(a) testing in all adults at least once.^19,37,38^ Support for a universal screening of adults by one-time measurement of plasma Lp(a) in primary prevention has recently been presented,^11^ but would constitute a formidable task for the medical health services if incorporated in routine clinical practice.^39^ Median plasma Lp(a) level has been observed to be higher in patients with prevalent atherosclerotic cardiovascular disease than in the general population and indicates an aggregation of individuals with high Lp(a) in cardiac care units.^10,40,41^ In light of this, the study finding that severely elevated Lp(a) (≥95th percentile) is almost 15-fold more common in FDR to indexes with a similar level than with low Lp(a) (<50th percentile) is important. Cascade screening initiated at a cardiac care unit from an index patient with coronary artery disease and elevated Lp(a) could potentially provide a valuable opportunity and an effective method for the healthcare service to identify FDR at high cardiovascular risk. The increased cardiovascular risk associated with severely elevated Lp(a) at a level of 200 mg/dL (approximately 430 nmol/L) has been reported to be similar to the risk observed in the autosomal dominant dyslipidaemia familial hypercholesterolaemia.^42,43^ Cascade screening of relatives to indexes with familial hypercholesterolaemia is an important and well-integrated part of best-practice patient care recognized by the International Atherosclerosis Society.^44,45^

### Strengths and limitations

A major strength of this study is the approach taken to evaluate the occurrence of major cardiovascular outcomes in FDR from a longitudinal perspective, with a focus on first-time events and premature disease. The cumulative incidences included adjustments for competing risk of non-cardiovascular death and for traditional cardiovascular risk factors at the end of follow-up. Outcomes are based on data from mandatory registries that are nationwide, comprehensive, and validated.^46^ Specifically, the linkage between indexes to FDR via the multigenerational register precludes selection bias of FDR.^18^ Together, the cohort size and the median follow-up of 19 years make the results robust and generalizable to other similar populations. The use of different laboratory methods to measure Lp(a) was evaluated in a sensitivity analysis, and we were able to exclude these from having significantly influenced the classification of indexes into Lp(a) strata.

The study has several limitations. The index cohort consisted of individuals who had their Lp(a) measured during routine clinic visits (2000–21) in Stockholm, Sweden. Since no regional or national guidelines were available for Lp(a) testing during this period, the indication for Lp(a) testing is not known and could therefore constitute a selection bias for the indexes. Lipoprotein(a) testing was also performed at all levels of patient care including primary, secondary, and tertiary care, adding to the complexity. However, the Lp(a) distribution in the indexes resembles that of the general population, indicating its representability. Further, although the inclusion in the cohort may be skewed towards patients at increased cardiovascular risk, the selection of indexes does not influence the association between elevated Lp(a) in indexes and cardiovascular outcomes in FDR.

First-degree relatives were evaluated for cardiovascular outcomes, but information on anthropometrics and lifestyle factors, including smoking, plasma lipids, and pharmacological treatment, was not available and could therefore constitute residual confounding. The evaluation of cardiovascular outcomes in indexes and FDR is also limited to individuals aged 35 to 69 years. The age span chosen, based on the availability of register data on outcomes (1 January 1987–31 December 2021), enables studies of individuals born between 1952 and 1987. While the age restriction provides robustness to the statistical comparisons between Lp(a) strata and minimizes the immortal time bias, it precludes all outcomes before the age of 35 years and leads to a slight underestimation of the actual risk.

## Conclusions

This is the first study describing the development of incident major cardiovascular outcomes in middle-aged FDR to indexes who had a Lp(a) test performed in the clinical routine. It demonstrates and quantifies a markedly increased risk for both female and male FDR to develop atherosclerotic cardiovascular disease, indicating the genetic predisposition to have elevated Lp(a) as a cardiovascular risk factor. In a future perspective, these data propose that cascade screening of first-degree family members to individuals with elevated Lp(a) could constitute a strategy of identifying individuals at heightened cardiovascular risk.

## Supplementary Material

ehaf677_Supplementary_Data

## Declarations

### Data Availability

The data that support the findings of this study are not publicly available. Access to data might be granted after review by the STRIREG steering committee.

### Pre-registered Clinical Trial Number

None supplied.

## References

[1] Kronenberg F, Mora S, Stroes ESG, Ference BA, Arsenault BJ, Berglund L, et al Lipoprotein(a) in atherosclerotic cardiovascular disease and aortic stenosis: a European Atherosclerosis Society consensus statement. Eur Heart J 2022;43:3925–46. 10.1093/eurheartj/ehac361 36036785 PMC9639807

[2] Nordestgaard BG, Langsted A. Lipoprotein(a) and cardiovascular disease. Lancet 2024;404:1255–64. 10.1016/S0140-6736(24)01308-4 39278229

[3] Larsson SC, Gill D, Mason AM, Jiang T, Back M, Butterworth AS, et al Lipoprotein(a) in Alzheimer, atherosclerotic, cerebrovascular, thrombotic, and valvular disease: Mendelian randomization investigation. Circulation 2020;141:1826–8. 10.1161/CIRCULATIONAHA.120.045826 32479194 PMC7614586

[4] Willeit P, Ridker PM, Nestel PJ, Simes J, Tonkin AM, Pedersen TR, et al Baseline and on-statin treatment lipoprotein(a) levels for prediction of cardiovascular events: individual patient-data meta-analysis of statin outcome trials. Lancet 2018;392:1311–20. 10.1016/S0140-6736(18)31652-0 30293769

[5] Szarek M, Bittner VA, Aylward P, Baccara-Dinet M, Bhatt DL, Diaz R, et al Lipoprotein(a) lowering by alirocumab reduces the total burden of cardiovascular events independent of low-density lipoprotein cholesterol lowering: ODYSSEY OUTCOMES trial. Eur Heart J 2020;41:4245–55. 10.1093/eurheartj/ehaa649 33051646 PMC7724642

[6] Seed M, Hoppichler F, Reaveley D, McCarthy S, Thompson GR, Boerwinkle E, et al Relation of serum lipoprotein(a) concentration and apolipoprotein(a) phenotype to coronary heart disease in patients with familial hypercholesterolemia. N Engl J Med 1990;322:1494–9. 10.1056/NEJM199005243222104 2139920

[7] Bjornson E, Adiels M, Taskinen MR, Burgess S, Chapman MJ, Packard CJ, et al Lipoprotein(a) is markedly more atherogenic than LDL: an apolipoprotein B-based genetic analysis. J Am Coll Cardiol 2024;83:385–95. 10.1016/j.jacc.2023.10.039 38233012 PMC7616706

[8] Bhatia HS, Wandel S, Willeit P, Lesogor A, Bailey K, Ridker PM, et al Independence of lipoprotein(a) and low-density lipoprotein cholesterol-mediated cardiovascular risk: a participant-level meta-analysis. Circulation 2025;151:312–21. 10.1161/CIRCULATIONAHA.124.069556 39492722 PMC11771346

[9] Raitakari O, Kartiosuo N, Pahkala K, Hutri-Kähönen N, Bazzano LA, Chen W, et al Lipoprotein(a) in youth and prediction of major cardiovascular outcomes in adulthood. Circulation 2023;147:23–31. 10.1161/CIRCULATIONAHA.122.060667 36440577 PMC9797445

[10] Patel AP, Wang M, Pirruccello JP, Ellinor PT, Ng K, Kathiresan S, et al Lp(a) (lipoprotein[a]) concentrations and incident atherosclerotic cardiovascular disease: new insights from a large national biobank. Arterioscler Thromb Vasc Biol 2021;41:465–74. 10.1161/ATVBAHA.120.315291 33115266 PMC7769893

[11] Kraaijenhof JM, Nurmohamed NS, Nordestgaard AT, Reeskamp LF, Stroes ESG, Hovingh GK, et al Low-density lipoprotein cholesterol, C-reactive protein, and lipoprotein(a) universal one-time screening in primary prevention: the EPIC-Norfolk study. Eur Heart J 2025;46:3875–84. 10.1093/eurheartj/ehaf209 40167249 PMC12517748

[12] de Boer LM, Wiegman A, Kroon J, Tsimikas S, Yeang C, Peletier MC, et al Lipoprotein(a) and carotid intima-media thickness in children with familial hypercholesterolaemia in the Netherlands: a 20-year follow-up study. Lancet Diabetes Endocrinol 2023;11:667–74. 10.1016/S2213-8587(23)00156-0 37487514

[13] MacDougall DE, Tybjaerg-Hansen A, Knowles JW, Stern TP, Hartsuff BK, McGowan MP, et al Lipoprotein(a) and recurrent atherosclerotic cardiovascular events: the US Family Heart Database. Eur Heart J 2025;46:4762–75. 10.1093/eurheartj/ehaf297 40331569 PMC12634116

[14] Kronenberg F, Utermann G. Lipoprotein(a): resurrected by genetics. J Intern Med 2013;273:6–30. 10.1111/j.1365-2796.2012.02592.x 22998429

[15] Perombelon YF, Soutar AK, Knight BL. Variation in lipoprotein(a) concentration associated with different apolipoprotein(a) alleles. J Clin Invest 1994;93:1481–92. 10.1172/JCI117126 8163653 PMC294162

[16] Coassin S, Kronenberg F. Lipoprotein(a) beyond the kringle IV repeat polymorphism: the complexity of genetic variation in the LPA gene. Atherosclerosis 2022;349:17–35. 10.1016/j.atherosclerosis.2022.04.003 35606073 PMC7613587

[17] Reeskamp LF, Tromp TR, Patel AP, Ibrahim S, Trinder M, Haidermota S, et al Concordance of a high lipoprotein(a) concentration among relatives. JAMA Cardiol 2023;8:1111–8. 10.1001/jamacardio.2023.3548 37819667 PMC10568442

[18] Andersson DP, Littmann K, Kindborg G, Eklund D, Sejersen K, Yan J, et al Relation among hypertriglyceridaemia, cardiometabolic disease, and hereditary factors-design and rationale of the Stockholm hyperTRIglyceridaemia REGister study. Eur Heart J Open 2024;4:oeae010. 10.1093/ehjopen/oeae010 38487365 PMC10937219

[19] Mach F, Baigent C, Catapano AL, Koskinas KC, Casula M, Badimon L, et al 2019 ESC/EAS guidelines for the management of dyslipidaemias: lipid modification to reduce cardiovascular risk. Eur Heart J 2020;41:111–88. 10.1093/eurheartj/ehz455 31504418

[20] Thomas PE, Vedel-Krogh S, Kamstrup PR, Nordestgaard BG. Lipoprotein(a) cardiovascular risk explained by LDL cholesterol, non-HDL cholesterol, ApoB, or hsCRP is minimal. J Am Coll Cardiol 2025;85:2046–51. 10.1016/j.jacc.2025.02.024 40266171

[21] Wang MC, Chang SH. Nonparametric estimation of a recurrent survival function. J Am Stat Assoc 1999;94:146–53. 10.1080/01621459.1999.10473831 24244058 PMC3826567

[22] The R Foundation for Statistical Computing . R core Team: A Language and Environment for Statistical Computing. In. Vienna, Austria: https://www.R-project.org/; 2024.

[23] Kamstrup PR, Benn M, Tybjaerg-Hansen A, Nordestgaard BG. Extreme lipoprotein(a) levels and risk of myocardial infarction in the general population: the Copenhagen city heart study. Circulation 2008;117:176–84. 10.1161/CIRCULATIONAHA.107.715698 18086931

[24] Emerging Risk Factors C, Erqou S, Kaptoge S, Perry PL, Di Angelantonio E, Thompson A, et al Lipoprotein(a) concentration and the risk of coronary heart disease, stroke, and nonvascular mortality. JAMA 2009;302:412–23. 10.1001/jama.2009.1063 19622820 PMC3272390

[25] Langsted A, Nordestgaard BG, Kamstrup PR. Elevated lipoprotein(a) and risk of ischemic stroke. J Am Coll Cardiol 2019;74:54–66. 10.1016/j.jacc.2019.03.524 31272552

[26] Ntaios G, Hart RG. Embolic stroke. Circulation 2017;136:2403–5. 10.1161/CIRCULATIONAHA.117.030509 29255121

[27] Roeters van Lennep JE, Tokgozoglu LS, Badimon L, Dumanski SM, Gulati M, Hess CN, et al Women, lipids, and atherosclerotic cardiovascular disease: a call to action from the European Atherosclerosis Society. Eur Heart J 2023;44:4157–73. 10.1093/eurheartj/ehad472 37611089 PMC10576616

[28] Chakraborty A, Chan DC, Ellis KL, Pang J, Barnett W, Woodward AM, et al Cascade testing for elevated lipoprotein(a) in relatives of probands with high lipoprotein(a). Am J Prev Cardiol 2022;10:100343. 10.1016/j.ajpc.2022.100343 35517871 PMC9062205

[29] Chakraborty A, Pang J, Chan DC, Ellis KL, Hooper AJ, Bell DA, et al Cascade testing for elevated lipoprotein(a) in relatives of probands with familial hypercholesterolaemia and elevated lipoprotein(a). Atherosclerosis 2022;349:219–26. 10.1016/j.atherosclerosis.2021.11.004 34862044

[30] Littmann K, Wodaje T, Alvarsson M, Bottai M, Eriksson M, Parini P, et al The association of lipoprotein(a) plasma levels with prevalence of cardiovascular disease and metabolic control Status in patients with type 1 diabetes. Diabetes Care 2020;43:1851–8. 10.2337/dc19-1398 31862789

[31] Kim K, Kim M, Han J, Jung H, Kim AR, Jun TJ, et al Impact of diabetes on risk of major adverse cardiovascular events associated with lipoprotein(a) levels in patients with established atherosclerotic cardiovascular disease. Eur J Prev Cardiol 2025;32:733–42. 10.1093/eurjpc/zwaf036 40066493

[32] Bajaj A, Damrauer SM, Anderson AH, Xie D, Budoff MJ, Go AS, et al Lipoprotein(a) and risk of myocardial infarction and death in chronic kidney disease: findings from the CRIC study (chronic renal insufficiency cohort). Arterioscler Thromb Vasc Biol 2017;37:1971–8. 10.1161/ATVBAHA.117.309920 28838919 PMC5620129

[33] Assmann SF, Pocock SJ, Enos LE, Kasten LE. Subgroup analysis and other (mis)uses of baseline data in clinical trials. Lancet 2000;355:1064–9. 10.1016/S0140-6736(00)02039-0 10744093

[34] Mehta A, Virani SS, Ayers CR, Sun W, Hoogeveen RC, Rohatgi A, et al Lipoprotein(a) and family history predict cardiovascular disease risk. J Am Coll Cardiol 2020;76:781–93. 10.1016/j.jacc.2020.06.040 32792075

[35] Tada H, Kojima N, Yamagami K, Takeji Y, Sakata K, Usui S, et al Association between lipoprotein (a) levels and coronary artery disease (CAD) among patients with or without CAD family history. J Lipid Atheroscler 2025;14:120–7. 10.12997/jla.2025.14.1.120 39911955 PMC11791416

[36] Wulff AB, Nordestgaard BG, Langsted A. Novel therapies for lipoprotein(a): update in cardiovascular risk estimation and treatment. Curr Atheroscler Rep 2024;26:111–8. 10.1007/s11883-024-01192-9 38311667

[37] Reyes-Soffer G, Ginsberg HN, Berglund L, Duell PB, Heffron SP, Kamstrup PR, et al Lipoprotein(a): a genetically determined, causal, and prevalent risk factor for atherosclerotic cardiovascular disease: a scientific statement from the American Heart Association. Arterioscler Thromb Vasc Biol 2022;42:e48–60. 10.1161/ATV.0000000000000147 34647487 PMC9989949

[38] Pearson GJ, Thanassoulis G, Anderson TJ, Barry AR, Couture P, Dayan N, et al 2021 Canadian cardiovascular society guidelines for the management of dyslipidemia for the prevention of cardiovascular disease in adults. Can J Cardiol 2021;37:1129–50. 10.1016/j.cjca.2021.03.016 33781847

[39] Kronenberg F, Bedlington N, Ademi Z, Geanta M, Silberzahn T, Rijken M, et al The Brussels international declaration on lipoprotein(a). Testing and Management. Atherosclerosis 2025;406:119218. 10.1016/j.atherosclerosis.2025.119218 40340180

[40] Berman AN, Biery DW, Besser SA, Singh A, Shiyovich A, Weber BN, et al Lipoprotein(a) and major adverse cardiovascular events in patients with or without baseline atherosclerotic cardiovascular disease. J Am Coll Cardiol 2024;83:873–86. 10.1016/j.jacc.2023.12.031 38418000 PMC12161880

[41] Nissen SE, Wolski K, Cho L, Nicholls SJ, Kastelein J, Leitersdorf E, et al Lipoprotein(a) levels in a global population with established atherosclerotic cardiovascular disease. Open Heart 2022;9:e002060. 10.1136/openhrt-2022-002060 36252994 PMC9577925

[42] Hedegaard BS, Bork CS, Kaltoft M, Klausen IC, Schmidt EB, Kamstrup PR, et al Equivalent impact of elevated lipoprotein(a) and familial hypercholesterolemia in patients with atherosclerotic cardiovascular disease. J Am Coll Cardiol 2022;80:1998–2010. 10.1016/j.jacc.2022.09.021 36396201

[43] Burgess S, Ference BA, Staley JR, Freitag DF, Mason AM, Nielsen SF, et al Association of LPA variants with risk of coronary disease and the implications for lipoprotein(a)-lowering therapies: a Mendelian randomization analysis. JAMA Cardiol 2018;3:619–27. 10.1001/jamacardio.2018.1470 29926099 PMC6481553

[44] Watts GF, Gidding SS, Hegele RA, Raal FJ, Sturm AC, Jones LK, et al International Atherosclerosis Society guidance for implementing best practice in the care of familial hypercholesterolaemia. Nat Rev Cardiol 2023;20:845–69. 10.1038/s41569-023-00892-0 37322181

[45] Nordestgaard BG, Chapman MJ, Humphries SE, Ginsberg HN, Masana L, Descamps OS, et al Familial hypercholesterolaemia is underdiagnosed and undertreated in the general population: guidance for clinicians to prevent coronary heart disease: consensus statement of the European Atherosclerosis Society. Eur Heart J 2013;34:3478–90. 10.1093/eurheartj/eht273 23956253 PMC3844152

[46] Ludvigsson JF, Andersson E, Ekbom A, Feychting M, Kim JL, Reuterwall C, et al External review and validation of the Swedish national inpatient register. BMC Public Health 2011;11:450. 10.1186/1471-2458-11-450 21658213 PMC3142234

