# Supplementary material for: Major cardiovascular events in first-degree relatives of individuals with elevated plasma lipoprotein(a): a registry-based cohort study
Source: Eur Heart J. 2025 Aug 31;47(26):3390–400. doi: 10.1093/eurheartj/ehaf677 (PMC13337222; doi:10.1093/eurheartj/ehaf677)
Supplement: ehaf677_Supplementary_Data [file ehaf677_supplementary_data.docx]

**SUPPLEMENTARY MATERIAL**

**Major cardiovascular events in first-degree relatives of individuals with elevated plasma lipoprotein(a): a registry-based cohort study**

**Authors: Gustav Kindborg, Daniel Eriksson Hogling, Henrike Häbel, Jane Yan, Teresa Hallerbäck, Örjan Lindhe, Emil Hagström, Daniel P. Andersson, Karin Littmann, and Jonas Brinck**

**Contents**

[Figure S1. Study flowchart according to Consolidated Standards of Reporting Trials (CONSORT) 2](#_Toc204611123)

[Figure S2. Lp(a) distributions in index cohort, in nmol/L (A) and mg/dL (B) 3](#_Toc204611124)

[Figure S3. Details on adjustment model covariates 4](#_Toc204611125)

[Figure S4. Assessment of proportional hazards assumption in indexes and FDR 5](#_Toc204611126)

[Figure S5. Cardiovascular death and coronary revascularization in FDR assessed between age 35–69 years stratified according to their index Lp(a) level 6](#_Toc204611127)

[Figure S6. Cumulative incidence, incidence rates and hazard ratios of major cardiovascular events in indexes assessed between age 35–69 years stratified according to their index Lp(a) level 7](#_Toc204611128)

[Figure S7. The pattern of concordance of plasma Lp(a) between individuals with different kind of first-degree relationships 9](#_Toc204611129)

[Table S1. Lp(a) laboratory methods and number of unique indexes assessed by each method 10](#_Toc204611130)

[Table S2. Lp(a) decile distributions in nmol/L and mg/dL between complete index cohort and cardiovascular outcome cohort 11](#_Toc204611131)

[Table S3. Sensitivity analysis for the different laboratory methods used to measure Lp(a) in indexes and their impact on classification into Lp(a) strata 12](#_Toc204611132)

[Table S4. Definition of cardiovascular outcomes and covariates according to International Codes of Diagnoses (ICD9, ICD10) and surgical intervention codes 13](#_Toc204611133)

[Table S5. MACE incidence rate for indexes assessed between age 35–69 years stratified according to their Lp(a) level 14](#_Toc204611134)

[Table S6. Incidence rates for coronary revascularizations (PCI, CABG) per 5-year interval in FDR 15](#_Toc204611135)

Author contributions…………………………………………………………………………16

# Figure S1. Study flowchart according to Consolidated Standards of Reporting Trials (CONSORT)

**
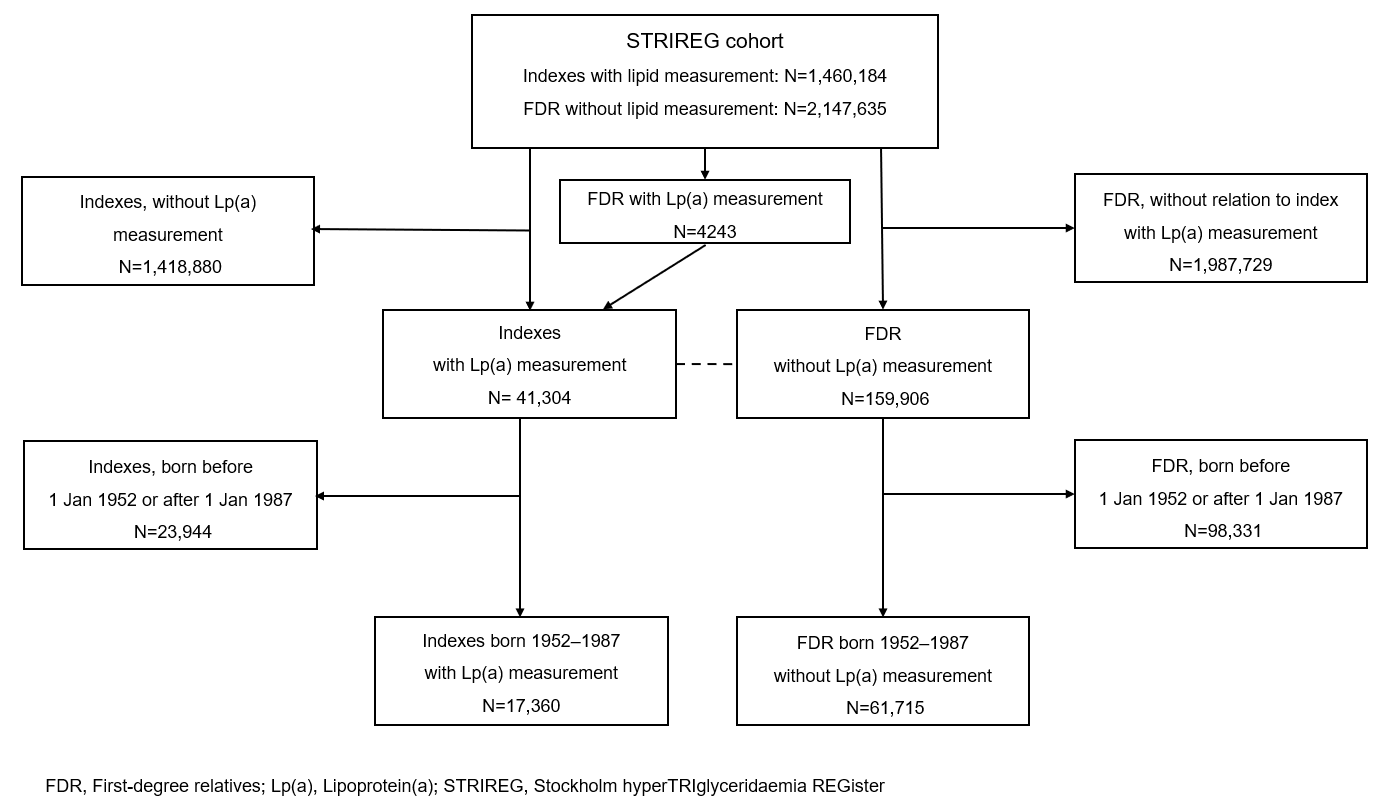
**

Figure S2. Lp(a) distributions in index cohort, in nmol/L (A) and mg/dL (B)

**
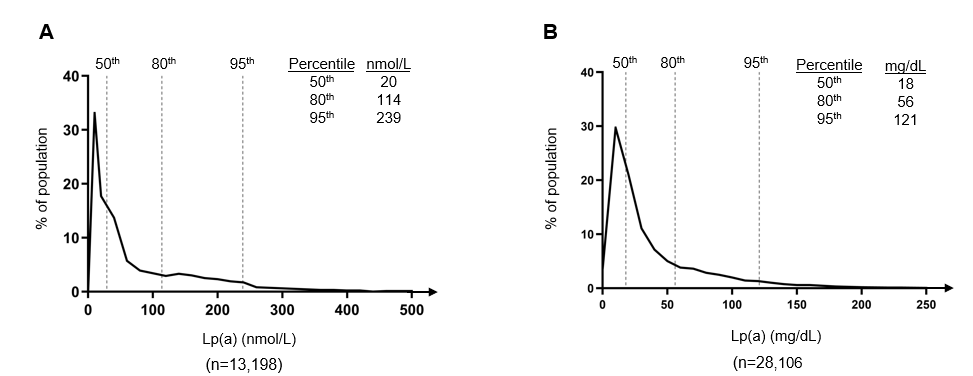
**

Lp(a), lipoprotein(a).

# Figure S3. Details on adjustment model covariates

The covariates entered into the model were chosen based on prior knowledge used in observational data, models derived from our prior work (Schubert et al. Eur Heart J 2024;**45**:4204-4215. <https://doi.org/10.1093/eurheartj/ehae576>) and from drawing a directed acyclic graph (DAG, see figure below). These covariates are age, sex, diabetes, hypertension, and chronic kidney disease (ever during follow-up).


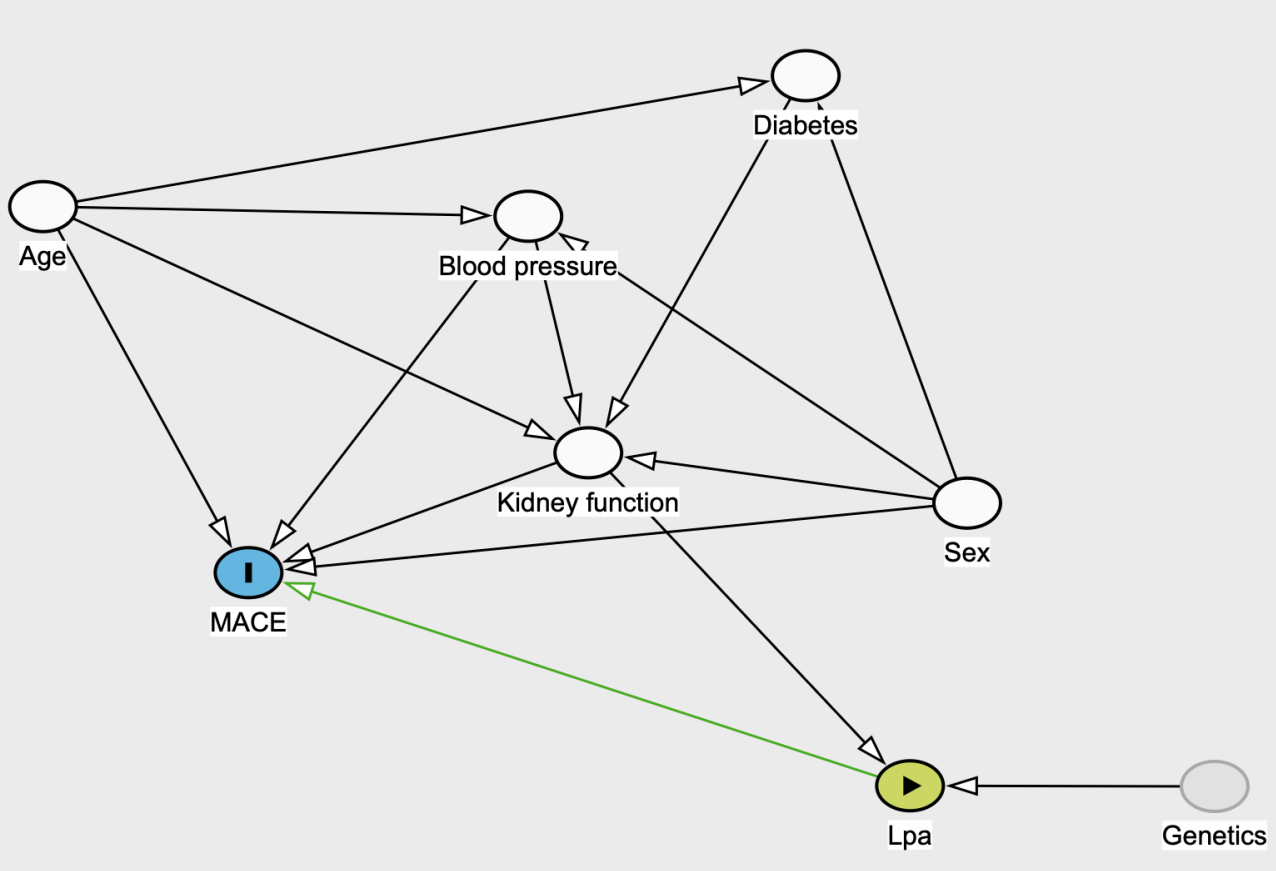


Lp(a), lipoprotein(a); MACE, major adverse cardiovascular events.

Directed acyclic graph (DAG) on confounders between lipoprotein (a) strata and major adverse cardiovascular events (MACE; cardiovascular death, myocardial infarction, ischaemic stroke, coronary artery revascularization). Green circle is exposure, blue circle is outcome, white circles are measured confounders, and grey circles are unmeasured confounders. MACE (major adverse cardiovascular event) is the composite outcome of all-cause mortality, myocardial infarction, or ischaemic stroke.

# Figure S4. Assessment of proportional hazards assumption in indexes and FDR

Index FDR


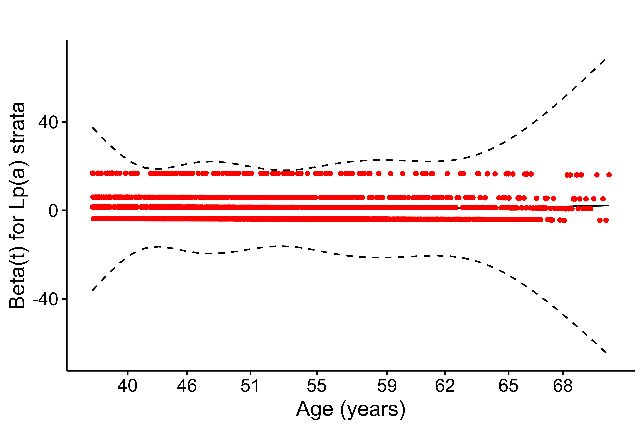

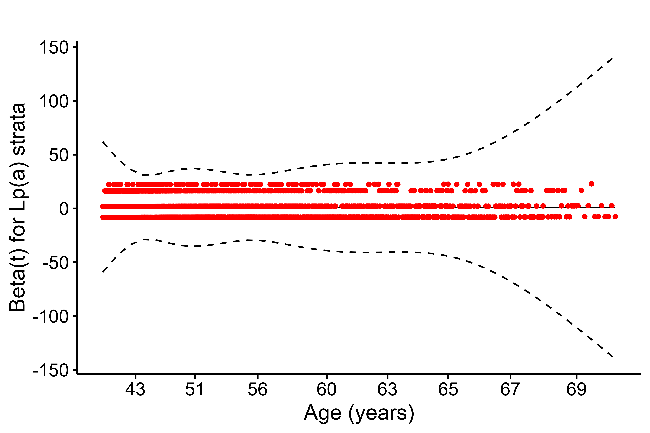


FDR, first-degree relative; MACE, major adverse cardiovascular events.

Schoenfeld residual plots for MACE to compare survival probabilities across groups.

# Figure S5. Cardiovascular death and coronary revascularization in FDR assessed between age 35–69 years stratified according to their index Lp(a) level

**
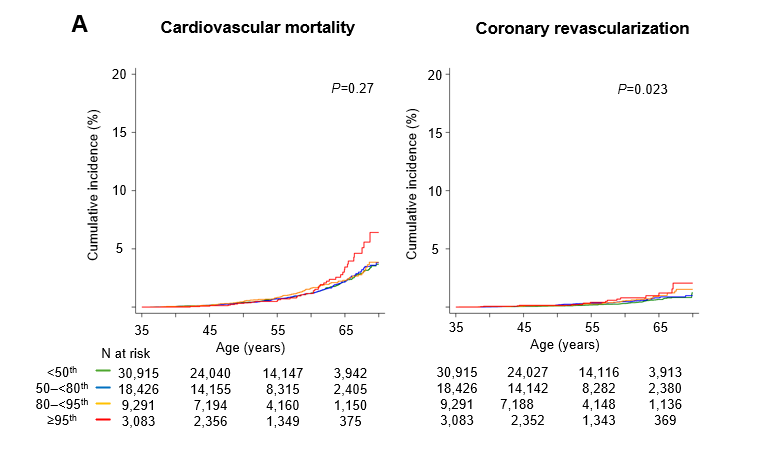
**

**
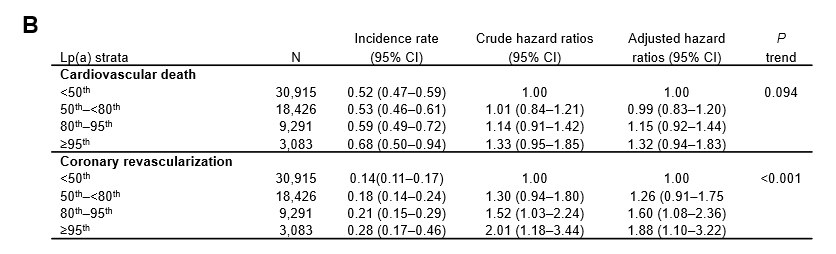
**

CI, confidence interval; FDR, first-degree relative; Lp(a), lipoprotein(a).

Competing risk-adjusted cumulative incidence (A), incidence rates and hazard ratios (B) of cardiovascular mortality and coronary revascularization in FDR aged 35–69 years stratified according to their indexes’ Lp(a) level. Incidence rates were calculated per 1000 person-years. Lowest Lp(a) strata was used as reference in the Cox regression model adjusted for sex, diabetes, hypertension and chronic kidney disease.

# Figure S6. Cumulative incidence, incidence rates and hazard ratios of major cardiovascular events in indexes assessed between age 35–69 years stratified according to their index Lp(a) level


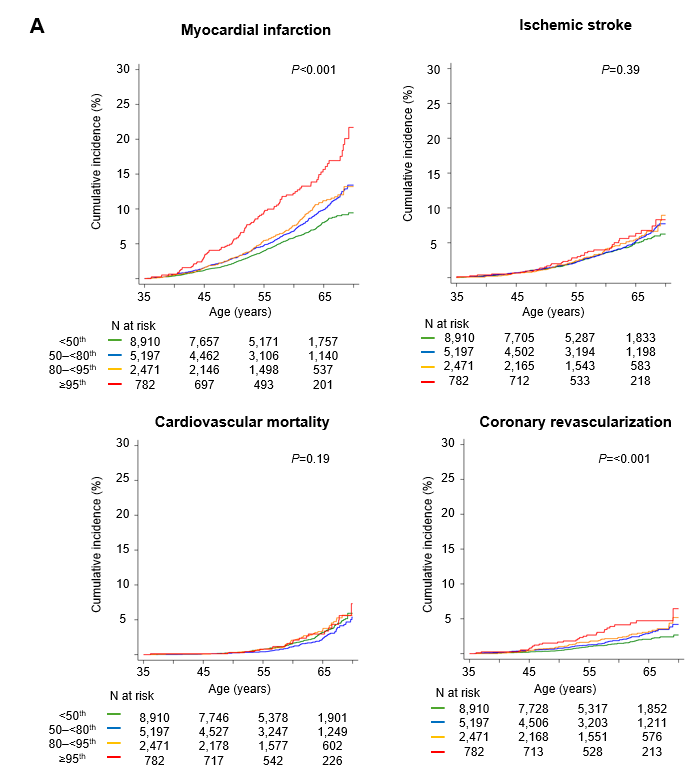


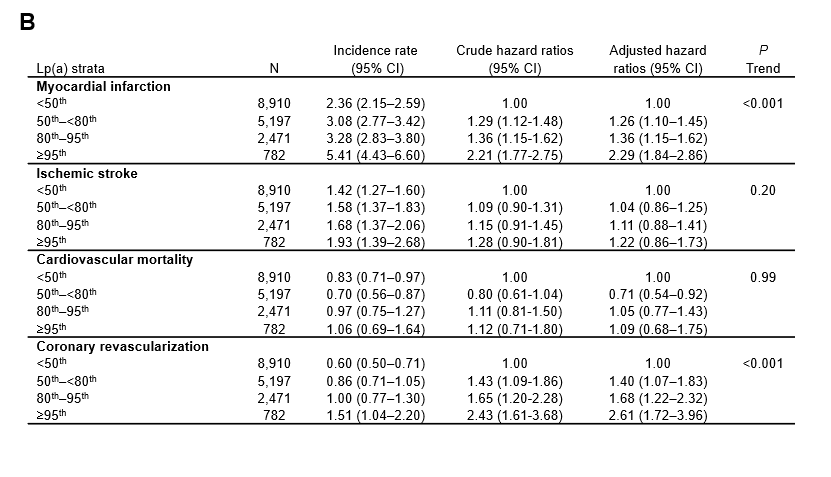


CI, confidence interval; FDR, first-degree relative; Lp(a), lipoprotein(a).

Competing risk-adjusted cumulative incidence (A), incidence rates and hazard ratios (B) of incident major cardiovascular events in indexes aged 35–69 years stratified according to their Lp(a) level. Incidence rates were calculated per 1000 person-years. Lowest Lp(a) strata was used as reference in the Cox regression model adjusted for sex, diabetes, hypertension and chronic kidney disease.

# Figure S7. The pattern of concordance of plasma Lp(a) between individuals with different kind of first-degree relationships


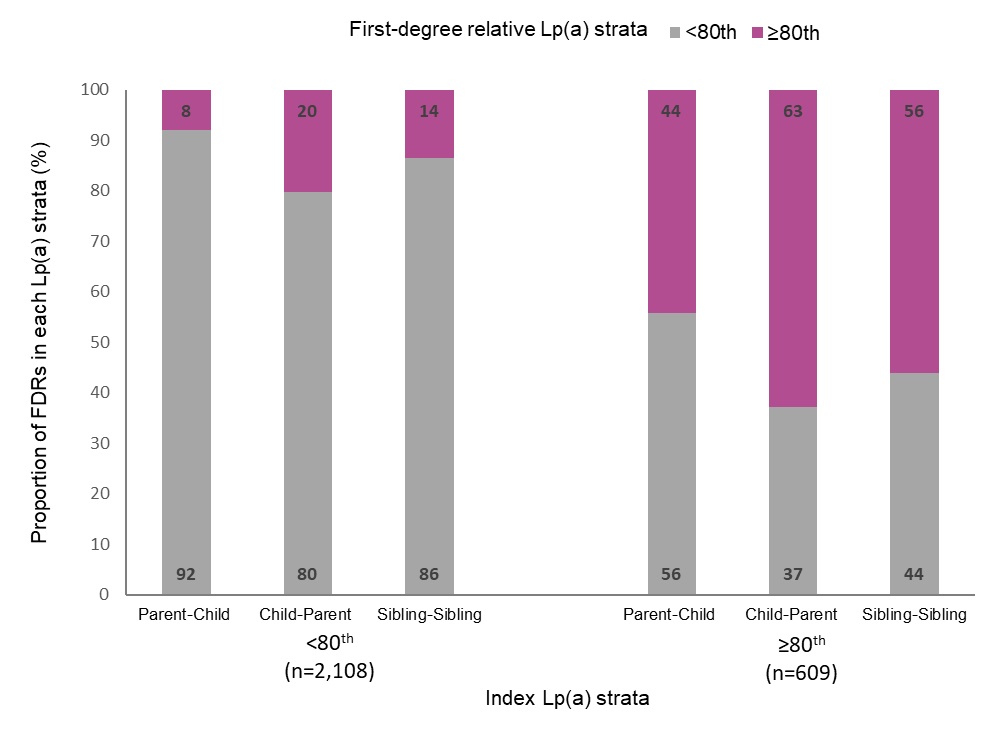


FDR, first-degree relative; Lp(a), lipoprotein(a).

# Table S1. Lp(a) laboratory methods and number of unique indexes assessed by each method

| **Internal laboratory analysis code** | **Reagent  (Time period used)** | **Instrument**  **(Time period used)** | **Laboratory** | **Matrix** | **Units** | **N** |
| --- | --- | --- | --- | --- | --- | --- |
| I: P LPA 01 | Lipoprotein (a), unknown  (2006) | Immage, Beckman Coulter  (2006) | Karolinska University Laboratory | Plasma | mass | 489 |
| II: P LPA 02 | Lipoprotein (a), unknown  (2006-2007)  LPAX Tina-quant Lipoprotein (a) (Latex) Roche Diagnostics  (2006-2015) | Immage, Beckman Coulter  (2006-2007)  Cobas 6000, Roche Diagnostics  (2006-2015) | Karolinska University Laboratory | Plasma | mass | 2954  8969 |
| III: P LPA 03 | LPA2 Tina-quant Lipoprotein (a) Gen 2 (Latex), Roche Diagnostics  (2014-2017)  LPA2 Tina-quant Lipoprotein (a) Gen 2 (Latex), Roche Diagnostics  (2018-2021) | Cobas 6000, Roche Diagnostics  (2014-2017)  Cobas 8000, Roche Diagnostics  (2018-2021) | Karolinska University laboratory, | Plasma | molar | 5528  7670 |
| IV: P-Lp(a) | Lp(a) FS DiaSys  (2000-2008)  Lp(a) FS* DiaSys  (2009-2018)  Lipoprotein (a), Siemens  (2018-2021) | Siemens Advia 1650 (2000-2008)  Siemens Advia 2400  (2009-2018)  Siemens Advia XPT and Siemens Atellica  (2018-2021) | Unilabs | Plasma | mass | 2204  2151  626 |
| V: S LPA D1 | Dakopatt a/s  (2000-2002)  LPAX Tina-quant Lipoprotein (a) (Latex) Roche Diagnostics  (2003-2005) | Hitachi 917  (2000-2002)  Modular P, Roche Diagnostics  (2003-2005) | Karolinska University Laboratory | Serum | mass | 4743  1295 |
| VI: S LPA K1 | Lipoprotein (a), unknown  (2000-2006) | Immage, Beckman Coulter  (2000-2006) | Karolinska University Laboratory | Serum | mass | 4189 |
| VII: S Apo Lp(a) | Lp(a) FS DiaSys  (2000-2008)  Lp(a) FS* DiaSys  (2009-2018)  Lipoprotein (a), Siemens  (2018-2021) | Siemens Advia 1650 (2000-2008)  Siemens Advia 2400  (2009-2018)  Siemens Advia XPT and Siemens Atellica  (2018-2021) | Unilabs | Serum | mass | 482  3  1 |

# Table S2. Lp(a) decile distributions in nmol/L and mg/dL between complete index cohort and cardiovascular outcome cohort

|  | **Lp(a) decile distribution (nmol/L) *** | | | | | | | | |
| --- | --- | --- | --- | --- | --- | --- | --- | --- | --- |
|  | **10^th^** | **20^th^** | **30^th^** | **40^th^** | **50^th^** | **60^th^** | **70^th^** | **80^th^** | **90^th^** |
| Complete index cohort  (n=13,198) | 3 | 6 | 9 | 13 | 20 | 32 | 58 | 114 | 187 |
| Cardiovascular outcome cohort (n=6829) | 3 | 5 | 9 | 13 | 20 | 33 | 58 | 112 | 188 |
|  | **Lp(a) decile distribution (mg/dL)** † | | | | | | | | |
|  | **10^th^** | **20^th^** | **30^th^** | **40^th^** | **50^th^** | **60^th^** | **70^th^** | **80^th^** | **90^th^** |
| Complete index cohort  (n=28,106) | 2 | 5 | 9 | 13 | 18 | 25 | 36 | 56 | 89 |
| Cardiovascular outcome cohort (n=10,531) | 2 | 5 | 9 | 12 | 16 | 23 | 33 | 52 | 83 |

Comparison of Lp(a) distributions was done by visual inspection and by the Kolmogorov-Smirnov test. Testing was performed by comparing individuals in the cardiovascular outcome cohorts and individuals in the complete cohorts excluding those in the cardiovascular cohort.

Lp(a), lipoprotein(a).

*p=0.35

†p<0.01

# Table S3. Sensitivity analysis for the different laboratory methods used to measure Lp(a) in indexes and their impact on classification into Lp(a) strata

| **Original strata**  **classification** | **New strata classification (%)** | | | |
| --- | --- | --- | --- | --- |
|  | <50^th^ | 50^th^–<80^th^ | 80^th^–<95^th^ | ≥95^th^ |
| <50^th^ (100%) | 95.6 | 4.4 | 0.0 | 0.0 |
| 50^th^–<80^th^ (100%) | 4.9 | 90.4 | 4.6 | 0.0 |
| 80^th^–<95^th^ (100%) | 0 | 3.1 | 89.9 | 7.0 |
| ≥95^th^ (100%) | 0.0 | 0.0 | 1.8 | 98.2 |

Lp(a), lipoprotein(a).

# Table S4. Definition of cardiovascular outcomes and covariates according to International Codes of Diagnoses (ICD9, ICD10) and surgical intervention codes

|  | **ICD-9** | **ICD-10** | **Surgical intervention code** | **Cause of death** |
| --- | --- | --- | --- | --- |
| Death |  |  |  |  |
| Non-cardiovascular death |  |  |  | Death excluding cardiovascular death |
| Cardiovascular death |  |  |  | Death from cardiovascular disease:  -ICD-9: 400-447  -ICD-10: I00-I99  (includes ICD-codes as underlying diagnosis and up to 5 contributory causes for fatal outcomes) |
| MACE, composite of:  *Cardiovascular death  *Myocardial infarction  *Ischaemic stroke  *Coronary  revascularization |  |  |  |  |
| Myocardial infarction | 410 | I21 |  |  |
| Ischaemic stroke | 433, 434 | I63, I64 |  |  |
| Coronary revascularization |  |  | 3080, 3065, 3066, 3068, 3092, 3105, 3127, 3158  FNA-H, FNJ-K, FNW |  |
| Hypertension | 401-405 | I10-I15 |  |  |
| Diabetes | 250 | E10-E14 |  |  |
| Chronic kidney disease | 285 | N18 |  |  |
| Peripheral artery disease | 440, 443, 250G, 440C | I70.0, I70.2, I73.9 |  |  |

MACE, major adverse cardiovascular events.

# Table S5. MACE incidence rate for indexes assessed between age 35–69 years stratified according to their Lp(a) level

| **Lp(a) strata** | **Incidence rate (95% confidence interval) per 1000 person-years** |
| --- | --- |
| <50^th^ | 4.38 (4.09–4.69) |
| 50^th^–<80^th^ | 5.17 (4.77–5.61) |
| 80^th^–95^th^ | 5.91 (5.29–6.60) |
| ≥95^th^ | 8.28 (7.03–9.74) |

MACE, major adverse cardiovascular events.

# Table S6. Incidence rates for coronary revascularizations (PCI, CABG) per 5-year interval in FDR

| **Time period** | **Exposure time, years** | **Events, n** | **Incidence rate, event/1000 person-years  (95% confidence interval)** |
| --- | --- | --- | --- |
| 1987–1991 | 25,298 | 3 | 0.118 (0.038–0.368) |
| 1992–1996 | 69,257 | 21 | 0.303 (0.198–0.465) |
| 1997–2001 | 119,458 | 10 | 0.084 (0.045–0.155) |
| 2002–2006 | 172,457 | 16 | 0.093 (0.056–0.151) |
| 2007–2011 | 219,948 | 27 | 0.123 (0.084–0.179) |
| 2012–2016 | 259,156 | 47 | 0.181 (0.136–0.241) |
| 2017–2021 | 291,527 | 76 | 0.261 (0.208–0.326) |
| **Overall** | **1,157,134** | **200** | **0.173 (0.150–0.198)** |

FDR, first-degree relative; PCI, percutaneous coronary intervention; CABG, coronary artery bypass graft surgery.

# Author contributions

All listed authors were involved in study conceptualization and/or interpretation of the study findings. Additionally, each author contributed to the drafting of the work or critical revision for important intellectual content. All authors approve the final version for publication and agree to be accountable for all aspects of the work.

GK Conceptualization: Equal

Investigation: Equal

Methodology: Equal

Project administration: Equal

Validation: Equal

Visualization: Equal

Writing – original draft: Lead

Writing – review & editing: Equal

DEH Conceptualization: Equal

Investigation: Equal

Methodology: Equal
Supervision: Supporting

Writing – review & editing: Equal

HH Data curation: Lead

Formal analysis: Lead

Methodology: Equal

Software: Lead

Visualization: Equal

JJ Data curation: Supporting

Formal analysis: Supporting

Methodology: Supporting

Software: Supporting

TH Conceptualization: Equal Investigation: Equal

Writing – review & editing: Equal

ÖL Conceptualization: Equal

Investigation: Equal

Writing – review & editing: Equal

EH Investigation: Equal

Writing – review & editing: Equal

DPA Investigation: Equal

Writing – review & editing: Equal

KL Conceptualization: Equal

Investigation: Equal

Methodology: Equal

Visualization: Equal

Supervision: Supporting

Writing – review & editing: Equal

J.B. Conceptualization: Equal

Funding acquisition: Lead

Investigation: Equal

Methodology: Equal

Project administration: Lead

Resources: Lead

Supervision: Lead

Validation: Equal

Visualization: Equal

Writing – original draft: Equal

Writing – review & editing: Lead
